# Issues for eHealth in Psychiatry: Results of an Expert Survey

**DOI:** 10.2196/jmir.6957

**Published:** 2017-02-28

**Authors:** Jennifer Nicholas, Kit Huckvale, Mark Erik Larsen, Ashna Basu, Philip J Batterham, Frances Shaw, Shahbaz Sendi

**Affiliations:** ^1^ Black Dog Institute University of New South Wales Australia Sydney Australia; ^2^ School of Psychiatry University of New South Wales Australia Sydney Australia; ^3^ Centre for Mental Health Research Australian National University Canberra Australia; ^4^ Affective Disorders Research Group, Center for Affective Disorders, Psychological Medicine Institute of Psychiatry, Psychology and Neuroscience King's College London London United Kingdom

**Keywords:** eHealth, mental health, technology adoption

## Abstract

**Background:**

Technology has changed the landscape in which psychiatry operates. Effective, evidence-based treatments for mental health care are now available at the fingertips of anyone with Internet access. However, technological solutions for mental health are not necessarily sought by consumers nor recommended by clinicians.

**Objective:**

The objectives of this study are to identify and discuss the barriers to introducing eHealth technology-supported interventions within mental health.

**Methods:**

An interactive polling tool was used to ask “In this brave new world, what are the key issues that need to be addressed to improve mental health (using technology)?” Respondents were the multidisciplinary attendees of the “Humans and Machines: A Quest for Better Mental Health” conference, held in Sydney, Australia, in 2016. Responses were categorized into 10 key issues using team-based qualitative analysis.

**Results:**

A total of 155 responses to the question were received from 66 audience members. Responses were categorized into 10 issues and ordered by importance: access to care, integration and collaboration, education and awareness, mental health stigma, data privacy, trust, understanding and assessment of mental health, government and policy, optimal design, and engagement. In this paper, each of the 10 issues are outlined, and potential solutions are discussed. Many of the issues were interrelated, having implications for other key areas identified.

**Conclusions:**

As many of the issues identified directly related to barriers to care, priority should be given to addressing these issues that are common across mental health delivery. Despite new challenges raised by technology, technology-supported mental health interventions represent a tremendous opportunity to address in a timely way these major concerns and improve the receipt of effective, evidence-based therapy by those in need.

## Introduction

The enormous personal, social, and financial burden caused by mental health problems is increasingly acknowledged. Mental health conditions are the leading cause of years lost to disability globally and account for 8.9% of global disability-adjusted life years [1]. A higher risk of suicide and increased medical comorbidity means that individuals with serious mental illness die up to 32 years earlier than the general population [2].

With the emergence of digital technologies, the landscape in which psychiatry operates has changed. There are now an expanded array of tools and resources at our disposal. Electronic health (eHealth) refers to a range of services that use information and communication technologies to improve human health. Examples include programs that deliver cognitive behavioral therapy to anonymous users over the Internet [3], remote assessment and treatment of patients using telecommunications and the Internet [4], and novel treatment interventions via virtual reality or serious games [5]. Recently, eHealth has expanded to include mobile health (mHealth), which aims to harness consumer-facing technologies such as smartphones and wearable devices to support health care.

In addition to direct delivery of services, eHealth technology can assist the administration and coordination of mental health care. For example, in the United Kingdom, the National Health Service (NHS) is integrating technology into primary care to allow patients to register with a general practitioner (GP), access health care records, and receive medical advice and information via their computer, smartphone, or tablet [6]. Allowing patients immediate access to private medical records via the Internet empowers consumers and may facilitate consistency of care.

However, while the promise of technology in mental health is high, delivery and uptake remains low. In the United States, only 20% of organizations involved in provision of behavioral support have adopted electronic records, compared with 60% of primary care organizations [7]. In Australia, an estimated 600,000 individuals with mild-moderate mental health disorders are potentially suitable for eHealth services, while only 25% currently receive any mental health care [8]. As yet, health system integration and the management, engagement, and prevention of mental health on a population scale remain unrealized possibilities. This is despite acceleration in consumer use of technology, with rapid adoption of platforms such as smartphones and new services for banking, commerce, travel, and social interaction.

There is a need to assess the reasons for this disparity. Is it just a matter of time [9], or are there barriers within mental health that will impede the deployment of technological solutions? Are the issues technological or do they arise from other sources? It is not clear what these problems, barriers, or concerns might be. Identification and discussion of possible issues may inform strategies to ensure the potential of technology for mental health is realized.

Taking advantage of a concentration of expertise drawn from across the mental health sector in Australia, we surveyed the audience of the “Humans and Machines: A Quest for Better Mental Health” conference about the key issues that need to be addressed to improve mental health using eHealth technologies. Based on audience responses, this paper aims to outline the perceived key issues and suggest ways to overcome these barriers.

## Methods

### Data Collection

Data were collected via a 1-question, cross-sectional, interactive survey using a convenience sample. In September 2016, the Black Dog Institute and University of New South Wales (UNSW) Australia hosted the “Humans and Machines: A Quest for Better Mental Health” conference in Sydney, Australia. The aim of the conference was to explore the interface between science, eHealth technologies, and human health and whether a physical face-to-face presence is required to provide quality mental health care. The survey question “In this brave new world, what are the key issues that need to be addressed to improve mental health (using technology)?” was presented to attendees at the end of the first session via the Poll Everywhere interactive data collection tool [10]. Attendees anonymously provided their free-text responses using the browser on their mobile devices. No limit was placed on the number of responses submitted by each individual.

### Data Analysis

Audience responses to the question were organized into key issue areas following guidelines for rigorous team-based approaches to decision making [11]. Two authors (JN and AB) independently generated 10 data-driven issue areas from the audience responses. Identification of issue areas was mostly inductive, allowing the analysis to be flexible and theory independent but guided by the research question [12]. Between the 2 coders, 11 unique issues were identified, of which 9 were identified by both coders. A third party (KB) with substantial experience with qualitative research resolved the difference in issue identification to provide a final list of 10 issues and any differences in individual response categorization in consultation with JN and AB. Issue importance was proxied by calculating the percentage of respondents that nominated each of the 10 issues identified.

## Results

A total of 94 individuals attended the “Humans and Machines: A Quest for Better Mental Health” conference. Speakers and audience members included a broad representation of senior staff spanning eHealth research, mental health professionals, health service providers, philanthropic organizations, and the health and technology industry. A total of 155 unique responses to the question were submitted by 66 audience members, from which 10 key issues that need to be addressed to improve mental health using technology were identified. The 10 issues identified were interrelated and are displayed in order of importance in Table 1.

**Table 1:** The 10 issues identified that need to be addressed to improve mental health (using technology) ranked in order of importance.

Issue identified	n (%)
Access to care	24 (36)
Integration and collaboration	15 (23)
Education and awareness	13 (20)
Mental health stigma	13 (20)
Data privacy	12 (18)
Trust	11 (17)
Understanding and assessment of mental health	11 (17)
Government and policy	10 (15)
Optimal design	9 (14)
Engagement	8 (12)

## Discussion

### Principal Findings

By combining the perspectives of a wide range of stakeholders drawn from a recent technology-focused conference, this opportunistic survey sought to provide a contemporary overview of shared priority issues that need to be considered if the potential of eHealth is to be realized in mental health. Rather than prespecify a technology, condition, or policy focus, the survey was intended to solicit the broadest range of opinions possible in order to understand the extent to which prevalent issues are technology-specific or, rather, represent an extension of known challenges in mental health generally. Validating this approach, of the identified issues, half (n=5) reflected pragmatic concerns of access, understanding, and attitudes to mental illness that extend beyond technology to stand as common barriers to improved mental health care. Technology cannot escape these issues. Yet there should also be considerable optimism in the potential for eHealth technologies to offer novel, substantive strategies to tackle these barriers to care.

Reflecting these dual notions of challenge and opportunity, the following discussion attempts to highlight how eHealth technology is shaped by and holds the potential to shape each identified issue. In addition to the service delivery themes identified above, the remaining issues fell into 2 further categories: structural issues surrounding mental health policy and services (n=2) and technology-specific issues (n=3).

### Issues Affecting Mental Health Service Delivery

#### Access to Care

Participants overwhelmingly highlighted the need to improve the timely access of mental health care by those in need. As well as general improvements in care access, reducing social inequalities in accessing mental health care was emphasized. Despite the range of effective strategies currently available to treat mental health conditions, too few individuals seek help. Projections indicate that improving service access among the two-thirds of Australians with a mental health disorder not receiving care would result in a 23% reduction in the burden of common mental disorders [13,14]. Addressing access to care involves consideration of barriers to care, many of which were identified by participants as issues requiring attention to improve mental health, including stigma, education, and health system integration and policy considerations. Additional proposed barriers to help seeking include a desire to handle the problem without outside help, distrust of mental health services [15], and concerns regarding cost, transport, time, and convenience [16].

Given the high rate of Internet access [17] and growing ownership of mobile devices [18] in both developed and developing settings, technology-supported interventions can address many of these identified barriers. Effective eHealth interventions are available in the form of unguided self-help [3,19] and can be accessed anonymously, minimizing the possibility of stigma. Such interventions provide around-the-clock access to evidence-based treatments, allowing timely access in response to symptoms and maximizing consumer convenience. eHealth interventions are, arguably, also able to provide more equitable access to health services, given their ability to be accessed remotely and at minimal to no cost to the user [20]. However, while technology may seem to address these barriers to help seeking with the potential to improve access to care, formal evaluation of the ability of technology-supported interventions to engage those otherwise not accessing care is required. Other factors that influence access to care, including trust discussed later, require consideration throughout design and development.

In aiming to address the issue of care access, technology-supported interventions must also consider scalability. Much has been made of the possibility for eHealth to have large-scale, population-based, system-wide implications for mental health [8]. However, populations are not homogeneous in terms of need, interest, or access to technology, all of which will mediate intervention success at scale.

Moreover, technology may itself generate inequalities in health service delivery and access. For example, technology-supported services often target younger adults based on their presumed affinity for technology [21]. Yet aptitude for and uptake of technological interventions may vary within generations as much as between. From divergent rates of device ownership [22] to the challenges of digital literacy [23], the same sociodemographic forces that shape health inequalities appear also to shape eHealth access. Beyond a need to design for diversity in technological experience, these findings recommend a particularly high bar in terms of demonstrating equity of access for any eHealth strategies that aim to replace existing mental health services.

#### Education and Awareness

Participants identified a need for further education in 2 mental health domains, mental health literacy and availability of eHealth interventions. Poor mental health literacy, defined as the lack of “knowledge and beliefs about mental disorders, which aid their recognition, management, or prevention” [15], has been identified as a key barrier to help seeking. Technology has the potential to be an important tool in educating the general population about mental health disorders with an aim to improve mental health literacy. The Internet has been successfully used to increase public education and awareness of mental and other health conditions [24,25], with resulting increases in intentions to seek help [24,25]. Further, the Internet is increasingly used as source of information about mental health, particularly among those with mental health problems [26].

Technology-supported mental health resources must consider how to disseminate information to the intended end-users, given eHealth interventions are currently not necessarily sought by consumers nor recommended by clinicians. Efforts for integration of eHealth into existing health care systems, discussed below, will increase clinician awareness of technology-supported interventions. Technology can support this effort—for example, the eMental Health in Practice (eMHPrac) initiative uses online continuing professional development–accredited learning modules supported by webinars, forums, and blogs to teach GPs, psychiatrists, and allied health professionals about eHealth, its efficacy, and role in routine care.

Similarly, there is a need to educate the public about eHealth options, which will support their role in clinical care, as patients will inquire about eHealth even when not suggested by their clinician. To this end, many online portals such as Beacon [27] and mindhealthconnect [28] seek to inform consumers about eHealth options and guide them to the most relevant evidence-based resources. However, given the instrumental role of Facebook, Twitter, and YouTube in the unprecedented success of the Ice Bucket Challenge in raising awareness (and funds) for amyotrophic lateral sclerosis in 2014 [29], the potential for technology, in particular social media, to increase mental health literacy and eHealth awareness is not currently fully realized.

#### Mental Health Stigma

A need to reduce the stigma associated with mental health conditions was evident in participant responses. Negative attitudes toward people (the self or others) with mental illness has been shown to be associated with lower intentions to seek help [30,31] and delayed or diminished recovery [32,33]. In an effort to reduce fear of stigma, online therapy today is often provided anonymously, minimizing the possibility that individuals will be identified as service users and avoiding the need to label individuals as having a mental health disorder. Yet while affordances offered by technologies such as anonymity and remote interaction may help to minimize stigma exposure, prevalent negative attitudes also slow technology adoption by reducing awareness of these new options, slowing help-seeking, and focusing technology discussion on physical health.

More radical is the potential for interventions that seek to modify stigmatizing attitudes directly, potentially at population-scale. In support of this potential, a Canadian media and social media campaign raised mental health awareness and reduced mental health stigma among young adults [34]. Internet-based interventions that provide either evidence-based therapy or psychoeducation have also been shown to reduce negative perceptions of mental illness and may increase the likelihood that individuals will seek professional help [25,35]. Further research should aim to identify other avenues for using technology to reduce stigma. For example, research has found that contact interventions (involving interpersonal contact with members of the stigmatized group) lead to greater reductions in stigma than providing psychoeducation alone [36]. Although video-based contact interventions may have less impact than face-to-face contact, the use of interactive games and other immersive technological experiences may provide an effective new avenue to combating the stigma of mental illness. The potential for social media to be harnessed as an advocacy tool to reduce stigma at a population level should also continue to be explored.

#### Understanding and Assessment of Mental Health

Shortcomings in the understanding of mental health conditions were highlighted by participants, including established difficulties in the field relating to assessment and classification, psychosocial determinants, and prevention [37]. Despite great advances in genetic and biological medicine, translation of these developments to the understanding and treatment of mental health disorders remains incomplete [2]. Further, individual differences in symptom presentation and disease courses, coupled with the subjective nature of mental health conditions, present challenges to the development of technology-supported resources for the understanding, treatment, and prevention of these disorders.

At the intersection of big data and psychiatry, consumer-facing technologies promise access to a vast array of personal and behavioral data. Given large enough datasets, previously hidden correlations—digital biomarkers—may yet emerge with the potential to better predict outcomes and further our understanding of mental health conditions. Current apps and wearables have the ability to passively collect data about activity (from Global Positioning System sensors and accelerometers), social connectedness (from Bluetooth connectivity, social media activity, and call and text logs), sleep/wake cycles (through light sensors and screen activation), and voice tone (from microphones).

However, both passively collected data and digital mental health resources must first be linked to clinically important outcomes and assessed using validated measures that accurately account for the continuum of mental health problems in the community. In mHealth, consensus regarding which patient-reported outcome measures are required to meaningfully assess app efficacy is needed [38].

Recent work has correlated passively collected objective data with clinically rated symptoms of depression and mania in bipolar disorder [39]. Further work with digital biomarkers will explore if data can predict changes in affective states, guide relapse prevention and clinical intervention, and ultimately inform the field about development [40], prodromes, and subclinical states [39]. Passively collected and user inputted data can also inform consumers and increase insight into their mental health, which is important for self-management.

Technology can also be used to assess risk. Digital footprints left by individuals’ online presence, in particular their social media use, have been used to assess risk of depression and suicide, highlighting the extension of technology to mental illness prevention [41].

#### Engagement

Participants emphasized prevalent challenges of engagement with mental health therapy. Nearly half of patients with a major depressive disorder drop out of therapy within 12 months [42]. Problems of engagement disproportionately affect young people [43] and those from minority groups, who are at least 40% more likely to discontinue treatment for a mood disorder, anxiety, or depression prematurely [44].

Technology-supported interventions have similar (or greater for open access resources) difficulties with engagement [45]. Although partly reflecting issues of stigma, confidentiality, and trust (identified as separate issues in this discussion), poor engagement encompasses additional factors that reduce the likelihood of meaningful initiation, participation in, and completion of therapy once enlisted in care. In addition to accidental causes of missed treatment, such as forgotten appointments or technical issues, mental health–specific factors include variable perceptions of treatment utility among patients and carers [46], prevalent delays around treatment [47,48], the alignment of available services with personal conceptions of mental health, and preferences for different styles and modes of therapy [46,49]. Poor engagement also extends to participation in mental health service design [50] and research [51].

However, technology-supported care has the promise of addressing the common mismatch between patient expectations and service capabilities, made possible by the ability to tailor content, motivational elements, and reminders to provide personalized therapy. Self-guided therapies, available through personal devices, can be initiated without delay in response to changes in condition state and pursued at times convenient to patients. Technology-based care can simultaneously support a spectrum of peer-, clinician-, community-, and agent-based interactions that offer genuine social support for some while guaranteeing autonomous self-care for others [3,52]. More broadly, technology platforms that integrate with social media have the potential to make positive contributions to discourse about mental health by sharing information about treatment and outcomes. Further, the enhanced ability to collect unobtrusive feedback and treatment participation data promises to accelerate future developments in the field.

### Structural Issues Surrounding Mental Health Policy and Services

#### Integration and Collaboration

Participants identified deficiencies in coordination of care and a perceived lack of interdisciplinary collaboration within the health care system as a challenge to technology-supported care. In particular, participants acknowledged the risk that new eHealth technologies perpetuate—or even extend—known challenges of service fragmentation that threaten continuity of care [53,54]. Indeed, the slow adoption of electronic health records in behavioral health settings continues to frustrate attempts to link services [7]. Many technology-supported interventions today sit independent of existing health care systems [8]. In addition to the potential complexity for decision making and patient choice arising from these new services, technology development models that do not emphasize clinical stakeholder involvement risk creating services that are a poor fit with referral and care pathways or back-office requirements, such as audit and billing.

Despite this, technology has the potential to address these challenges through better information sharing, better use of information contained in health records, and more effective communication between professionals, patients, and carers. The success of this cohesive picture will hinge on the successful and timely introduction of technology into existing systems of care.

One promising integration approach is the stepped care model in which technology-supported interventions are incrementally introduced as part of a continuum of therapies of differing intensity targeting a specific condition [8]. Such interventions have proven success in treating mild-moderate anxiety and depression [19] and release traditional resources to serve individuals with more severe symptoms [8]. It is projected that stepped care will increase quality of care for consumers and lower mental health costs by providing cost-effective care to those with mild-moderate disorders while reducing the burden on face-to-face services and increasing workforce participation.

Systemic change will require not only government support but, critically, buy-in from organizations involved in mental health delivery and leadership from clinical champions [55]. Increased awareness of technological mental health interventions among consumers and clinicians aided by mental health professional education and training will be important [8]. Further, shared information technology infrastructure and successful deployment of electronic health records will be necessary to ensure continuity of care from technology-supported to in-person services [8]. Substantive interdisciplinary collaboration that incorporates consumer perspectives will also become increasingly important as the dependencies between services and systems grows [56].

#### Government and Policy

For the field to flourish, participants suggested governments will need to develop frameworks and policies to encourage innovation and technology within health services. Recent findings indicate that government-facilitated access to electronic medical records through patient portals increases consumer involvement in health care and improves health outcomes [57,58]. The Australian government has recognized the potential and cost effectiveness of technology in mental health care service delivery [59], supporting the e-mental health record and the eMHPrac initiative designed to promote online mental health resources in primary care [60]. Dedicated centers of excellence for eHealth research are also supported, as well as organizations providing services directly to the public which receive approximately 275,000 combined unique website visits each month [8].

In addition to mentioning an enabling role for policy makers, participants also highlighted the pressing need for timely governance of emerging technologies characterized by a rapid pace of change. For example, although regulation concerning the development of health apps now exists in some health economies, notably in the United States [61], the scope of these guidelines is restricted to diagnostic and therapeutic categories that commonly exclude mental health. This patchy regulatory coverage allows anyone to develop and deploy an app for mental health through commercial app stores without an evidence base. Clinical assessments of mental health apps have highlighted a lack of evidence-based content and minimal demonstrations of efficacy [62,63]. While this may not affect consumer uptake, clinicians are understandably wary of recommending apps to support treatment in the absence of quality guarantees, slowing integration into care. Beyond apps, the emerging scope for technology-supported population-scale digital mental health prevention and health promotion campaigns will open up new issues around data governance and ethics that lie outside existing governance frameworks. Market-initiated solutions such as clearing houses, development guidelines, and quality checklists may have a role [64], but effective regulation of medical technologies has historically relied on government intervention.

### Technology-Specific Issues

#### Data Privacy

Participants highlighted the need to safeguard the privacy of identifiable, sensitive health information collected by eHealth services. Secure data storage and the choice to remain anonymous were considered necessities when dealing with health data. Further, given the ability to passively collect an unprecedented diversity and volume of personal and behavioral data through smartphone apps, responses emphasized informed, user-controlled data collection.

Unfortunately, there is often considerable opacity regarding data collection and processing in technological interventions, illuminated only by privacy policies that are often long and difficult to understand. The availability of a privacy policy is not, however, a comprehensive solution for understanding the privacy implications of a technological intervention. A study of app privacy found the majority of policies did not actually focus on the app concerned [65]. Furthermore, privacy policies do not necessarily reflect what happens “inside the black box,” as 78% of accredited apps in the now defunct NHS Health Apps Library uploaded data which had not been disclosed to users [66].

Although these limitations may not factor in user decisions on whether or not to use an eHealth platform, they nevertheless reflect noncompliance with relevant privacy regulations, which hinder the possibility of ethical, informed decisions regarding the use of specific platforms. To assist consumers, as well as ensuring the enforcement of privacy regulations, the provision of simplified user-friendly privacy information has been proposed, akin to the recent overhaul in the presentation of nutritional information [67].

#### Trust

Reflecting wider conceptions of distrust of mental health services as a barrier to care [15], participants identified multiple dimensions to the concept of trust in the use of technology for mental health, including ethical data collection and analysis and the need for its responsible use. The costs of breaching trust were considered catastrophic. Organizations involved in data collection and analysis or delivering eHealth interventions need to have the trust of users. Further, users, including mental health professionals, also need to be able to trust the technologies. Medical practitioners have been found to “see data as costs, risks, and liabilities” [68].

Similarly, it is important to consumers to trust that data will be used for the public good [69]. Context is critical for these perceptions of trust, with considerations around why and how data will be used contributing to whether or not data feels right or feels wrong [70]. Therefore, transparency in data collection purposes, access, and uses should be emphasized.

There must also be trust that the technologies and techniques used for data analysis are secure and effective and will provide accurate identification of mental health symptoms or risk for mental health problems. Kennedy [71], drawing on the work of Theodore Porter, argues that quantification apparently reduces the need for interpersonal trust through the appearance of objectivity in data analysis techniques but that reliance on numbers can increase distrust if errors are made. This highlights the importance of evidence and accuracy in the use of eHealth interventions and data analysis in mental health. Therefore, it is paramount that the evidence-base for the wide range of technological interventions in mental health is developed, as is the case for online interventions for common mental health disorders [3,19]. Further, certification or accreditation for eHealth and mHealth programs could enhance consumer trust of these resources.

#### Optimal Design

Participants highlighted uncertainties about the optimal design of technology-supported mental health interventions. Some reflected longstanding thematic concerns for eHealth applicable not only to mental health, such as how best to translate therapeutic principles to a technology-based medium while retaining clinical effectiveness, how to identify patient groups most likely to benefit from technology-supported care, and how to appropriately tailor both platform (whether Web, app, or social media) and design to ensure usability and acceptability among target users [72,73]. While a perceived benefit of eHealth interventions is their ability to address varied experience and personal risk factors as well as cultural norms in diverse populations, this increases the complexity and cost of design.

Lessons learned in other disciplines may guide design considerations. Recent work in health promotion has highlighted the potential to design complex interventions for behavior change using discrete building blocks, contributing to theory-building, and maximizing likely effectiveness [74]. Strategies such as user-centered and participatory design, which emphasize substantive involvement of target users throughout intervention development, can simultaneously refine intervention focus while eliminating potential usability barriers [56]. New evaluation strategies, which are better suited to both iterative improvements in intervention designs and the fast past of technology change, will also be needed [75].

However, there is work to be done to optimize these techniques for the design of mental health interventions. For example, any theory seeking to maximize user interaction, whether through motivational elements or gameplay, must also be compatible with psychological theory guiding therapy. As a result, effective design requires not only technical proficiency from software developers, but commensurate skills among clinical staff to understand the conceptual basis behind concepts such as serious games and translate these in ways that achieve specific desired outcomes such as improved adherence [44] and are compatible with evaluation [76].

### Limitations

The limitations of this paper require acknowledgment. The survey was conducted using a convenience sample of attendees at a conference convened to discuss the interface between science, technology, and human health and the potential role of technology in providing quality mental health care. Given a convenience sample and a common interest in technology, attendee perspectives around the challenges of mental eHealth may not be fully representative, particularly of stakeholders who have made a principled choice *not* to use eHealth technologies. However, attendees were also experts in eHealth with research and organizational roles where a balanced understanding of the issues could be reasonably expected.

A further limitation is that consumers were not represented among conference attendees, limiting the issues identified to those important from a service provision perspective. It is widely acknowledged that consumers and service providers traditionally hold different views on mental health care challenges and priorities [77], and thus an important and varied perspective is not represented in these results. Future research should address this gap and aim to understand the issues in the introduction of technology in mental health service delivery perceived by consumers.

Finally, the frequency-of-elicitation method used to assess relative importance may be an imperfect proxy for participant views. Factors other than importance that may have influenced participant submissions include accidental omission and perceptions that a topic might have already have been submitted by others. It is therefore possible that, given a forced choice method, a different ranking would have emerged. As a result, while the consistency of themes that emerged (despite the diversity of participant backgrounds) strengthens the convergent validity of the issues considered as a set, the rank order should be interpreted with caution.

### Conclusions

Current mental health service provision has failed to engage a large number of those in need. Many of the issues identified by attendees of the “Humans and Machines: A Quest for Better Mental Health” conference directly relate to barriers to care, including access to care, stigma, education, engagement, integration, and government and policy. Priority should therefore be given to addressing these issues that are common across mental health delivery. Despite new challenges, technology-supported mental health interventions represent a tremendous opportunity to overcome these issues but only if they are actively considered during design and development.

Several studies have shown that the use of technology in mental health care is acceptable and at times preferable to consumers, with convenience, cost, and anonymity listed among its advantages [78]. Indeed, in some cases, the use of technology for mental health care has been largely consumer-driven (e.g., the use of apps).

However, to fulfill this potential, an integrated, coordinated approach is needed to establish a role for eHealth services within existing health care systems and increase awareness of these services among consumers and clinicians. This requires commitment from all stakeholders, including research, clinical practice, regulators, and governments to support the role of technology in mental health. Research has established the effectiveness of a range of e-mental health services, but an emphasis on implementation science is essential to ensuring the successful scaling of digital health interventions. Clinician awareness and training programs are vital to inform and support the role of eHealth in routine practice and to guarantee consumers are directed to appropriate technological interventions. Critically, while government recognition of the benefits of eHealth within the health care system is important to the delivery of eHealth programs, this recognition may be meaningless without sustained funding to maintain eHealth services and for continuing development to ensure that health interventions keep pace with emerging technology.

Without a concerted effort to translate research into policy and practice to address the barriers described here, the adoption of technology into mental health care will inevitably be slowed. However, the greater failure will be to miss the potential, offered by technology, to address in a timely way major concerns of access, stigma, and engagement that stand as active barriers to participation in mental health care and the receipt of effective, evidence-based therapy by those most in need.

## Abbreviations

eMHPrac: eMental Health in Practice
GP: general practitioner
NHS: National Health Service
UNSW: University of New South Wales
